# Transformation of *Botrytis cinerea *by direct hyphal blasting or by wound-mediated transformation of sclerotia

**DOI:** 10.1186/1471-2180-11-266

**Published:** 2011-12-21

**Authors:** Shahar Ish - Shalom, Aviva Gafni, Amnon Lichter, Maggie Levy

**Affiliations:** 1 Postharvest Science of Fresh Produce, The Volcani Center, ARO, Israel; 2 The Department of Plant Pathology and Microbiology, The Robert H. Smith Faculty of Agriculture, Food and Environment, The Hebrew University of Jerusalem, Rehovot, Israel

**Keywords:** *Botrytis cinerea*, sclerotium, *Sclerotinia sclerotiorum*, transformation, 'Bim-Lab', electroporation

## Abstract

**Background:**

*Botrytis cinerea *is a haploid necrotrophic ascomycete which is responsible for 'grey mold' disease in more than 200 plant species. Broad molecular research has been conducted on this pathogen in recent years, resulting in the sequencing of two strains, which has generated a wealth of information toward developing additional tools for molecular transcriptome, proteome and secretome investigations. Nonetheless, transformation protocols have remained a significant bottleneck for this pathogen, hindering functional analysis research in many labs.

**Results:**

In this study, we tested three different transformation methods for *B. cinerea*: electroporation, air-pressure-mediated and sclerotium-mediated transformation. We demonstrate successful transformation with three different DNA constructs using both air-pressure- and sclerotium-mediated transformation.

**Conclusions:**

These transformation methods, which are fast, simple and reproducible, can expedite functional gene analysis of *B. cinerea*.

## Background

*Botrytis cinerea *is a haploid necrotrophic ascomycete which is known as a major pathogen responsible for 'grey mold' disease in more than 200 plant species [1-3]. It attacks aboveground plant organs, and is a major pathogen during post-harvest storage due to its exceptional ability to grow, develop and attack produce at low temperatures. The high impact of diseases caused by *B. cinerea *has triggered a wide scope of molecular research in recent years, resulting in the sequencing of two *B. cinerea *strains. This has generated a wealth of information on the genome of this fungus (http://www.broadinstitute.org/annotation/genome/botrytis_cinerea/Home.html;http://urgi.versailles.inra.fr/index.php/urgi/Species/Botrytis)[4]. Along with genomic research, the development of additional tools, such as transcriptome, proteome and secretome analysis, has yielded additional information on the mechanisms underlying *B. cinerea *pathogenicity. These methods have filled in some of the gaps in our knowledge but unlike model organisms such as *Neurospora crassa *[5], functional analysis remains a significant bottleneck. The first requirement for functional analysis is a robust and high-throughput transformation protocol. However, the existing protoplast-based and Agrobacterium-mediated transformation methods [6-11] are complex and time-consuming; moreover, protoplast preparation is tedious and requires an enzyme cocktail whose consistency between batches is unknown. Here we describe two alternative protocols--direct hyphal transformation by blasting [12] and wounding-mediated transformation of sclerotia--both fast, simple and reproducible methods which might improve functional analysis in *B. cinerea *and other sclerotium-forming fungi.

## Methods

### Fungal cultures and growth conditions

*B. cinerea *isolate BO5.10 was maintained on potato dextrose agar (PDA, 39 g/L, BD Biosciences, Franklin Lakes, NJ, USA) amended with 250 mg/L chloramphenicol (Sigma-Aldrich, St. Louis, MO, USA) at 22-25°C for 7 to 10 days on 90-mm diameter Petri dishes. Conidia were harvested with purified water (resistivity > 18.2.CM; Millipore Milli-Q system) containing 0.001% (w/v) Triton X-100 (Sigma-Aldrich). The number of conidia was counted under a light microscope, at 400× magnification. Selection media consisted of Gamborg B5 pH 5.7 containing 3.16 g/L Gamborg B5 powder with vitamins (Duchefa, Haarlem, The Netherlands), 0.7 g/L of sodium nitrate (Sigma-Aldrich) and 3% (w/v) glucose amended with 50-250 μg/mL hygromycin B (Hyg) (Roche, Basel, Switzerland) and 15 g/L agar or PDA plates, pH 7.1, amended with 20 μg/mL phleomycin (Phleo)(InvivoGen, California, USA).

### Preparation of the DNA constructs

**Figure 1:**
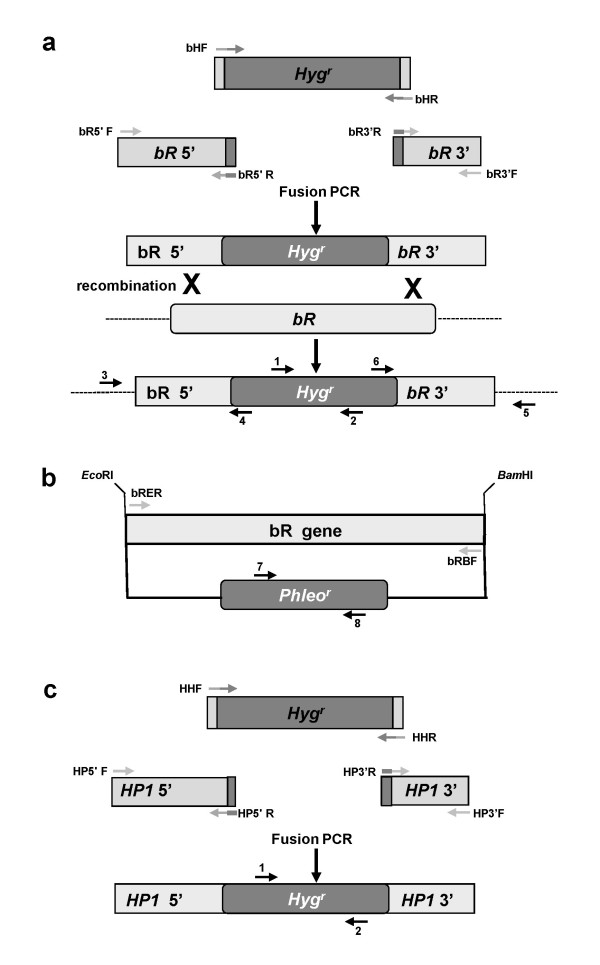
**Constructs for transformation of *B. cinerea***. (a) *bR *knockout construct is based on the work of Shafran and colleagues [13] and contains two flanking regions of the *bR *gene (bR 3' and bR 5') and in between the Hyg^r ^cassette as selection marker. Homologous recombination with genomic DNA is presented (dashed lines are genomic flanking regions next to *bR *gene). (b) The *bR *gene was cloned into the pBC-Phleo plasmid between the *Eco*RI (upstream) and *Bam*HI (downstream) restriction sites upstream to the Phleo^r ^cassette. (c) The *HP1 *knockout construct is composed of two flanking regions of the gene and in between a Hyg^r ^cassette as selection marker. The relative location of primers which were used to verify transformation is marked by arrows and numbers (detailed in Methods, primer sequences are listed in Table 1).

The bacterio-Rhodopsin (*bR*) (BC1G_02456.1) knockout construct (Figure 1a) was based on a modified Gateway vector (Invitrogen, Gaithersburg, MD, USA)[13]. The regions which flank the *bR *gene (BC1G_02456.1) are present on both sides of the Hyg^r ^cassette. The upstream 420-bp fragment (bR 5') was amplified using primers: bR5'F AGATGGGGCGGCTGGGTA and bR5'R AGATC-CCACTATCCTATCA. The downstream 418-bp flanking region (bR 3') was amplified using the primers bR3'F TAGTCGCGAACGATGTGAAG and bR3'R GAACACATCGTCCGTTTCCT. The middle region of the hygromycin resistance cassette (Hyg^r^) (1832 bp) was amplified using the primers bRHF GGGG-ACAACTTTGTATAGAAAAGTTGGCGGCCGCCACAAAGACCTCTCGCCTTT and bRHR GGGGACAACTTTGTATAATAAAGTTGGCGGCCGCCCGACTCCCAACTCG-ACTAC. Fragments were joined together by PCR in three stages as previously described [12].

The pBC-*bR*^Phleo ^construct (Figure 1b) was generated by cloning the *bR *gene (1068 nt) using primers: bRBF: AGCCTCGTCCTGTACAACTATAGGATCCCATCCCA-CAACATAACTCT and bRER: TTAACTGTACTCCTATCCTATACTTAAGATACTTTTCGGTTAGAGCGGATG into the pDES-Phleo vector [14] between the *Eco*RI (upstream) and *Bam*HI (downstream) restriction sites.

The third construct, knocked out in hypothetial protein 1 (*HP1*) (BC1G_14370.1), was generated by fusion of three PCR fragments (Figure 1c) [15]. The upstream fragment of *HP1 *(524 bp) was amplified by the primers: HP5'F AGTGTTCAACGAGCTCCA; HP5'R AGGTGAGTGTTGCGGCTAGT and the downstream flanking region (83 bp) was amplified using primers: HP3'F GGATAAAGAACAGCTAATCT and HP3'R ACTAGCCGCAACACTCACCT. The Hyg^r ^cassette (3728 bp) was amplified from pCT74 [16] using primers HHF: AGGTGAGTGTTGCGGCTAGTGCACTGCTCTGCTGTCTCTGAAGCTGGTCC G, and HHR: ATCAGTTAACGTGGATAAAGAACA. After sequencing, the PCR fragments were joined to the Hyg^r ^fragment by PCR with the nested primers (HP5'F and 3'HR TTCAATATCAGTTAACGTCGACCTCGTTCTGGATATGGAGGA and 5'HF CCAGTTGAATTGTCTCCTCCAGTCGACGTTACTGGTTCCCGGT and HP3'R) as described previously [15].

### Protoplast preparation

Protoplasts were prepared as previously described by Noda and colleagues [17] with some modifications. Conidia from a well-sporulated plate were harvested and used to inoculate 100 mL of liquid malt medium containing (per L): 5 g glucose, 15 g malt extract (Bacto Malt Extract, BD Biosciences), 1 g casein peptone (Sigma-Aldrich), 1 g yeast extract (BD Biosciences), 1 g casamino acids (Sigma-Aldrich). The culture was shaken overnight at 150 rpm at 18 to 22°C. The developing mycelium was collected on a Nytex membrane and the membrane was washed with 60 mL sterile water followed by two washes with 0.6 M cold KCl buffer (AnalaR, Leicestershire, England) containing 50 mM CaCl_2 _(Amerco, Reno, NV, USA). The washed mycelium (1.2 to 1.5 g) was transferred into a 50-mL Erlenmeyer flask with 10 mL filter-sterilized protoplast solution containing 0.4 mg/mL lysing enzymes (Sigma-Aldrich, cat no. L-1412-5G) suspended in KCl buffer. The suspension was shaken for 1 to 2 h at 85 rpm and 28°C and generation of protoplasts was monitored by light microscope. The protoplasts were generated from germinating conidia, broken hyphae or both sources together and were separated from the original tissue over a 60-mesh Nytex membrane (Sigma-Aldrich). Protoplasts were further purified by two centrifugations (1485*g*, 4°C, 10 min) and the pellet was washed by gentle resuspension in 10 mL KCl buffer, centrifuged, resuspended in the residual buffer and transferred into a new pre-cooled tube. The number of protoplasts was adjusted to 10^8 ^cells/mL.

### Electroporation

The electroporation protocol was adapted from [18], with some modifications, and used on either protoplasts or germinated conidia. Protoplasts were prepared as described above and washed with cold electroporation buffer containing 1 mM N-2-hydroxyethlpiperazine-N'-2-ethanesulfonic acid (HEPES, Sigma-Aldrich), 50 mM mannitol (Sigma-Aldrich), pH 7.5. Conidia were incubated in malt medium for 4 h at 25°C, centrifuged (835*g*, 4°C) and then washed with cold electroporation buffer and their concentration was adjusted to 10^8 ^conidia/mL. Aliquots of protoplasts or germinated conidia (100 μL) were dispensed in cold electroporation cuvettes (Bio-Rad, Hercules, CA, USA) and 2.5 to 10 μg DNA was added. The electroporation was performed with a 'Gene Pulser' (Bio-Rad) operated at 1.4 kV, 800 W and 25 μF. After application of the electrical pulse, the conidia or protoplasts were transferred to regeneration medium containing (per L purified sterile water): 145.7 g mannitol (Sigma-Aldrich), 4 g yeast extract, 1 g soluble starch and 16 g agar (Difco Laboratories, Detroit, MI, USA). After 10 h, an overlay of 10 mL HM medium consisting of: 1% (w/v) malt extract, 4% glucose, 0.4% (w/v) yeast extract, 125 mg Na_2_HPO_4_, 320 mg NH_4_C1, 180 mg MgSO_4 _7H_2_0, 13 mg CaC1_2 _2H_2_O, 4 mg FeC1_3 _6H_2_O, 250 mg Na_2_SO_4_, 1100 mg MES, 1300 mg HEPES and 1.5% agar, pH 5.5 with 50 μg/mL of hygromycin B (Hyg), was poured onto the plates. Colonies appeared after 4 to 5 days and were transferred to Gamborg B5 solid medium with 50 μg/mL Hyg or PDA medium supplemented with 20 μg/mL Phleo.

### Transformation of sclerotia

For sclerotium transformation, *B. cinerea *or *Sclerotinia sclerotiorum *sclerotia were collected from mature colonies grown on PDA plates for 10 days or more at 22°C or 18°C, respectively. Sclerotia were disinfected by three washes with 1% sodium hypochlorite, followed by three washes with sterilized purified water. The sclerotia were dried between washes on sterile Whatman filter paper in a biological hood and were completely dried prior to transformation. The dried sclerotia were wounded by generating a hole in the middle of the sclerotia (without penetrating through) with a sterile needle (21G) followed by four applications at 30-s intervals of 5 μL DNA solution (a total of 0.5 to 2 μg) or sterile purified water, both supplemented with 0.01% (v/v) Silwet L-77 surfactant (Agri-Turf Supplies, Santa Barbara, CA, USA). After 10 to 15 min, the solution was fully absorbed and sclerotia were placed on water-agar plates which were then incubated for 1 to 2 days at 22°C. At this stage, sclerotia were transferred to solid selective media. When vacuum was added to this procedure, the sclerotia were transferred, after wounding, into a 1.5-mL polypropylene tube and covered with DNA solution (0.05 μg/mL) or water as a control, both supplemented with 0.01% Silwet L-77 surfactant. The tubes were placed in a Speedvac (Heto Lab Inc., Laurel, MD, USA) and vacuum was applied for 20 min. The growing hyphae were then transferred onto new plates containing Gamborg B5 solid medium and 50 μg/mL Hyg. Viable colonies were transferred to new plates (55-mm Petri dish) with increasing concentrations of Hyg, up to 250 μg/mL in steps of 50 μg/mL, or PDA medium supplemented with 20 μg/mL Phleo that was increased to 60 μg/mL in 10 μg/mL steps.

### Transformation by hyphal blasting

The hyphal-blasting procedure was adopted from Levy and colleagues [12] with some modifications. PDA plates were inoculated in the center with 20 μL of spore suspension (10^7 ^spores) and then incubated at 22°C for 24 to 48 h until the colony diameter was in the range of 2 to 2.5 cm. Before use, blast cassettes were cleaned by immersion in soap and water, followed by five washes with sterile purified water, disinfection with 70% ethanol and drying in a biological cabin. For the blasting procedure, a 'Bim-Lab' instrument (Bio-Oz, Yad Mordehai, Israel) was used. The instrument was adjusted to the manual setting at a pressure of 2 bars, and the 'gun' was set at a height of 15 cm above the Petri dish. Cassettes were loaded with 0.5 to 3 μg of the DNA solution or sterile purified water diluted with 0.01% Silwet L-77 surfactant. The cassette containing the DNA was connected to the gun and the DNA was blasted over the edge of the colony mycelium four to five times at 10-s intervals until drops were fully dispersed over the plate. Plates were then incubated for 20 to 24 h and 10 plugs from the perimeter of the colony were transferred to Gamborg B5 solid medium plates supplemented with 50 μg/mL Hyg.

### Analysis of transformants

The stability of the strains in all of the above methods was verified by five transfers of the colony edges onto new solid Gamborg B5 medium with increasing concentrations of Hyg (from 50 to 250 μg/mL) or PDA medium supplemented with 20 μg/mL Phleo that was increased up to 60 μg/mL in 10 μg/mL steps and then five subsequent transfers onto PDA plates without the selection.

For DNA extraction, *B. cinerea *mycelium was grown in 20 mL Gamborg B5 liquid medium for 3 days and harvested by filtration over three layers of sterile 3 MM paper discs. Freshly harvested (100 to 200 mg) or lyophilized (10 to 20 mg) mycelia were added to 2-mL tubes with a volume of glass beads (Sigma-Aldrich, 200-300 μm) equivalent to 100-200 μL, 700 μL breaking buffer (2% Triton X-100, 1% w/v SDS, 100 mM NaCl, 10 mM Tris-HCl pH 8.0, 1 mM EDTA, all from Sigma-Aldrich) and 500 μL chloroform:isoamyl alcohol (24:1, v/v). Tubes were sealed and vortexed for 7 to 10 min, at a speed of 7-8 in a Genie 2 vortex (Scientific Industries, Inc., New York, NY, USA) and then centrifuged for 10 min at maximum speed (Eppendorf 5415 D). The supernatant (500 μL) was amended with 50 μL of 3 M sodium acetate pH 5.2 and 1 mL of 96% ethanol. Tubes were then incubated for 45 min at -20°C and DNA was precipitated by centrifugation (10 min, maximum speed). The DNA was washed with 70% ethanol and centrifuged for 5 min at 10,500*g*. The pellet was dried for 1 h in a biological hood and suspended in 100 μL TE (10 mM Tris HCl pH 8.0, 0.1 mM EDTA) or sterile ultra-purified water. DNA extraction from *S. sclerotiorum *was performed by fast extraction from mycelial plugs using NaOH as described by Levy and colleagues [12].

To verify transformation, we performed PCR analyses on DNA extracted from putative transformants using Hyg^r ^(Hyg F and Hyg R) and Phleo^r ^(Phleo F and Phleo R) cassette primers (Table 1). For verification of knockout by homologous recombination, a 480-bp fragment was amplified with primer bR-gen 5'F, which is located in the 5' upstream genomic region of the *bR *gene and is not present in the 5' fragment of the *bR *construct, and primer bR-Hyg 5'R from the Hyg cassette; a 590-bp fragment was amplified by primer bR-gen 3'F which is located in the 3' downstream genomic region of the *bR *gene and is not present in the 3' fragment of the *bR *construct, and primer bR-Hyg 3'R which is located at the 3' end of the Hyg cassette.

**Table 1:** Primer details for the PCR analysis of transformants

**No**.	Name	Sequence	Fragment size (bp)
1	Hyg F	CGACGTTACTGGTTCCCGGT	
2	Hyg R	GCGGGCACGTTAACTGAT	550
3	bR-gen 5'F	ACAAGACCTCTCGCCTTT	
4	bR-Hyg 5'R	AGGTCGGAGACGCTGTCGAA	480
5	bR-gen 3'F	ATGCAGCTTGGGCTGTTCAG	
6	bR-Hyg 3'R	CGACTCCCAACTCGACTA	590
7	Phleo F	GGGGACAAGTTTGTACAAAAAAGCAGGCT	
8	Phleo R	GGGGACCACTTTGTACAAGAAAGCTGGGT	1020
			

All PCR analyses were performed in 0.2-mL tubes containing PCR reagent (ReddyMix^®^, Thermo Fisher Scientific Inc., Surrey, UK) with 5 pmol of primers, 12.5 to 25 ng template DNA and sterile purified water to a final volume of 20 μL. PCR was carried out on a T-gradient PCR instrument (Biometra, Goettingen, Germany). Activation of the enzyme was carried out at 95°C for 5 min followed by denaturation for 45 s at 94°C, annealing at 62°C for 45 s, elongation at 70°C for 45 s for 30 to 40 cycles, and 10 min of elongation at 70°C. PCR products were analyzed on a 1 to 2% agarose gel according to their size and stained with 'Safeview' (G108 SafeView™ Nucleic Acid Stain, Applied Biological Materials Inc., Richmond, Canada).

## Results

### Protoplast-mediated transformation by electroporation

Three different DNA constructs were used for transformation of *B. cinerea *(Figure 1). The *bR *knockout construct (Figure 1a) was based on a modified Gateway vector according to Shafran and colleagues [13] (see Methods). This construct was used with all transformation methods. Protoplasts generated from germinating conidia or broken hyphae were used for electroporation experiments: the few colonies that slowly recovered from electroporation did not survive the Hyg selection.

### Sclerotium-mediated transformation

**Figure 2:**
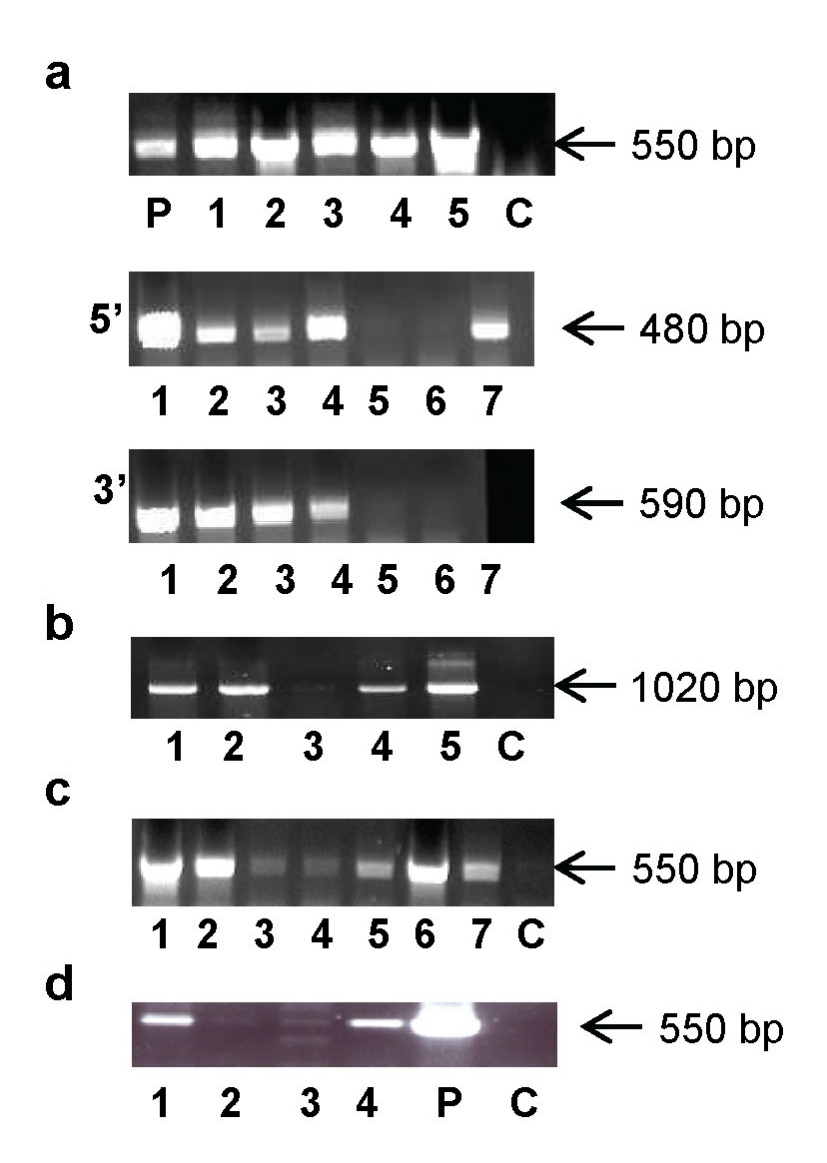
**PCR analyses of transformants of *B. cinerea*. and *S. sclerotiorum***. (a) A fragment of the Hyg^r ^cassette (550 bp) was amplified by primers 1 and 2 from five different *bR *knockout strains (1-5). A 480-bp fragment was amplified by primer 3 which is located in the 5' upstream genomic region of the *bR *gene and by primer 4 in the Hyg cassette (5'), and a 590-bp fragment was amplified by primer 5 which is located in the 3' downstream genomic region of the *bR *gene and primer 6 which is located at the 3' end of the Hyg cassette (3'); P is the positive control of the *bR *knockout construct (plasmid DNA). (b) A fragment of the Phleo^r ^cassette (1020 bp) was amplified by primers 3 and 4 from four different *bR *complementation strains (1-4). C is the negative control of the WT strain. (c) A fragment of the Hyg^r ^cassette (550 bp) was amplified by primers 1 and 2 from the *HP1 *transformants (1-7). C is the negative control of the WT strain. (d) A fragment of the Hyg^r ^cassette (550 bp) was amplified by primers 1 and 2 from four transformants of *S. sclerotiorum *(1-4). P is the positive control of the Hyg^r ^cassette (plasmid DNA) and C is the negative control of the WT strain (primers sequences are listed in Table 1).

Both *B. cinerea *and *S. sclerotiorum *are known to produce sclerotia--melanized and condensed hyphal structures which are able to survive harsh environmental conditions in nature. Sclerotia can be readily collected from mature (over 10 days old on a Petri dish) cultures and preserved dry under ambient conditions. The possibility of transforming sclerotia is therefore very appealing. Sclerotia were collected from mature culture (> 10 days old), disinfected, wounded with a needle, and DNA supplemented with surfactant Silwet L-77 was introduced by pipetting directly onto the wound. Silwet L-77 was chosen because it reduces surface tension more than most surfactants and has been found to greatly enhance bacterial entry into relatively inaccessible plant tissues in plant transformation [19,20]. In an experiment with the *bR *knockout construct, 45 sclerotia yielded 21 (46%) Hyg-resistant and PCR-positive transformants (Table 2, Figure 2a), and 13 (62%) of these strains were identified as knockout strains by PCR of the Hyg cassette with the flanking region of *bR *genomic DNA (Figures 1a and 2a). These results demonstrated the feasibility of sclerotium-mediated transformation.

**Table 2:** Transformation with the *bR *knockout construct

	Blast	Sclerotia	Electroporation
Experimental material	Mycelium^1^	Sclerotia	Cells^2^
Quantity per experiment^3^	10	45	3 x10^6^
Transformants^4^	39%	46%	0
Putative knockouts^5^	54%	62%	0

^1^On PDA plates.

^2^Protoplasts generated from broken hyphae, germinating conidia or both.

^3^Number of plates used for blasting. Ten plugs were excised from each plate resulting in 100 isolates subjected to Hyg selection.

^4^Verified by Hyg selection and PCR.

^5^Homologous recombination verified by PCR and sequencing.

To further verify the reproducibility of this method, we introduced the pBC-*bR*^Phleo ^complementation cassette, which contains the *bR *gene and the Phleo selection marker (Phleo^r^) on pBC-Phleo [14], into the *B. cinerea bR *knockout strain (Figure 1b). The vector was introduced into sclerotia in its native circular structure. The experiment included 120 sclerotia resulting in recovery of 65 Phleo-resistant and PCR-positive isolates (54%) (Table 3). A third construct for knockout of *HP1 *was generated by fusion PCR [15] (see Methods) (Figure 1c). It was introduced into 20 sclerotia, resulting in three transformants (15%) (Figure 2c, Table 4).

**Table 3:** Transformation with the pBC-bR^Phleo ^construct

	Blast	Sclerotia
Experimental material	Mycelium^1^	Sclerotia
Quantity per experiment^2^	10	120
Transformants^3 ^(%)	34%	54%

^1^On PDA plates.

^2^Number of plates used for blasting. Ten plugs were excised from each plate resulting in 100 isolates subjected to Phleo selection.

^3^Verified by Phleo selection and PCR.

**Table 4:** Transformation with the *HP1 *knockout construct

	Blast	Sclerotia
Experimental material	Mycelium^1^	Sclerotia
Quantity per experiment^2^	4	20
Transformants^3^	30%	15%

^1^On PDA plates.

^2^Number of plates used for blasting. Ten plugs were excised from each plate resulting in 40 isolates subjected to Hyg selection.

^3^Verified by Hyg selection and PCR.

To test whether sclerotium-mediated transformation can be extended to other sclerotium-producing fungi, a linear plasmid containing a Hyg^r ^cassette [12] was introduced into sclerotia of *S. sclerotiorum*, resulting in 5 to 10% transformation efficiency as verified by PCR analysis (Figure 2d) and application of vacuum resulted in a higher number of transformants (data not shown). Other knockout constructs were also successfully introduced into *S. sclerotiorum *with high efficiency (unpublished data). These results suggest that transformation of sclerotia is a viable approach, while it remains to be determined if the efficiency of transformation is construct-dependent [21].

### Direct hyphal transformation

Another transformation approach which was extensively tested was direct hyphal transformation using a high-pressure air pulse obtained from a 'Bim-Lab' instrument to bombard and transform mycelia [12]. Unlike conventional bombardment, this method employs a DNA solution that contains a surfactant rather than solid particles such as tungsten or gold. The mixture of DNA construct and surfactant is blasted over the periphery of the growing colony onto the hyphal tips during the early stages of growth. Blasting conidia or germinating conidia with the *bR *knockout construct did not yield any transformants. However, when blasting was performed on a young colony (24-48 h post-inoculation), we obtained 66% putative transformants, while older colonies (72-96 h post-inoculation) produced only 25% putative transformants. In terms of efficiency, five experiments with the *bR *knockout construct resulted in 50 colonies yielding 39% transformants (Table 2), and 21 (54%) of them were identified as knockout strains by PCR of the Hyg cassette with the flanking region of *bR *genomic DNA (Figures 1a and 2a). Therefore, we used young colonies for further experiments with the pBC-*bR*^Phleo ^vector and *HP1 *construct. Transformation with pBC-*bR*^Phleo ^resulted in 60 Phleo-resistant colonies, 34% of which were PCR-positive strains (Table 3), while transformation with the *HP1 *construct yielded an average of 3 transformants from 10 colonies (30%) (Table 4).

## Discussion

Since protoplast-based and Agrobacterium-mediated transformation methods are complex and time-consuming, we set out to develop new and simple transformation methods for *B. cinerea*. We tested different transformation methods, two of which were based on published transformation protocols (electroporation and blasting) and one which is a newly developed method and is based on wounding-mediated transformation of sclerotia.

Electroporation did not yield any results despite repeated attempts under various conditions. In addition, there is no published protocol for *B. cinerea *and other labs that have tried this method have not reported positive results. Of the other two methods described, transformation of sclerotia was efficient (15-50%), easy to perform, required no dedicated instruments or reagents, and colonies appeared after a relatively short time. A significant advantage of this method is the possibility of storing sclerotia for long periods but obviously, it can only be used on strains and species which form sclerotia. The second method, bombardment of DNA with high-pressure air blasted directly onto the growing hyphal tips, also demonstrated good efficiency (30-40%) and took only a short time, as has also been shown for *S. sclerotiorum *[12]. Unlike conventional bombardment, this method employs a DNA solution that contains a surfactant, which may assist in DNA penetration into the cells [17,18], rather than solid particles such as tungsten or gold [22]. However, it should be noted that this method requires a specialized instrument. Both methods required small amounts of DNA construct, which can be a significant advantage in terms of cost and throughput, and both methods demonstrated facilitated, high efficiency gene targeting (50-60%).

One possible explanation for the positive results with the sclerotium and blast methods is the fact that they impose minimal stress on the cells. In contrast, the electroporation method requires diversion of metabolism to cell-wall regeneration and membrane recovery, and both of these processes may result in a significant stress response. We believe that these reproducible and reliable transformation procedures will increase the efficiency of transformation, will simplify and improve our ability to resolve gene function in this important phytopathogen, and can be easily calibrated for additional fungi.

## Conclusions

In this study we describe two alternative protocols--direct hyphal transformation by blasting and wounding-mediated transformation of sclerotia, which are fast, simple and reproducible and might improve functional analysis in *B. cinerea *and other sclerotium-forming fungi. Future studies will show if these transformation methods have wide applicability for improved gene-targeting efficiency and if they can replace current methodologies.

## Abbreviations

PDA: potato dextrose agar; bR: bacterio-Rhodopsin; HP1: hypothetical protein 1; Hyg: hygromycin B; Phleo: phleomycin/Zeocin.

## Competing interests

The authors declare that they have no competing interests.

## Authors' contributions

ML and AL designed the experiments. SIS and AG performed the experiments. ML, AL and SIS wrote the manuscript. All authors read and approved the final manuscript.

## Author information

^1^Postharvest Science of Fresh Produce, The Volcani Center, ARO, Israel; ^2 ^The Department of Plant Pathology and Microbiology, The Robert H. Smith Faculty of Agriculture, Food and Environment, The Hebrew University of Jerusalem, Israel
